# Optimization of extraction and enrichment process of cannabidiol from industrial hemp and evaluation of its bioactivity

**DOI:** 10.3389/fpls.2025.1495779

**Published:** 2025-02-07

**Authors:** Junkai Wu, Xiaomeng Zhang, Xiaoqing Liu, Zunlai Sheng, Jianping Hu, Feiyan Zhang

**Affiliations:** ^1^ School of Pharmacy, Quanzhou Medical College, Quanzhou, China; ^2^ College of Veterinary Medicine, Northeast Agricultural University, Harbin, China; ^3^ Heilongjiang Key Laboratory for Animal Disease Control and Pharmaceutical Development, Northeast Agricultural University, Harbin, China

**Keywords:** industrial hemp, cannabidiol, extraction process, enrichment process, bioactivity evaluation

## Abstract

**Introduction:**

The *Cannabis Sativa* L., a perennial dioecious herb renowned for its industrial applications, serves as the source of hemp. Cannabidiol (CBD), a non-psychotropic compound derived from industrial hemp, has garnered considerable interest due to its promising therapeutic potential.

**Methods:**

The extraction parameters for CBD from industrial hemp were optimized using the Box-Behnken design and response surface methodology (RSM). The purification process involved characterizing the penetration and desorption profiles of CBD on HPD-100 resin. The *in vitro* antibacterial activity was assessed by determining the minimum inhibitory concentration (MIC) against *Staphylococcus aureus* and *Escherichia coli*. Antioxidant properties were evaluated using DPPH and ABTS assays, as well as an iron-reducing ability test.

**Results:**

After optimization, the extraction rate of CBD reached 0.26 ± 0.02%. The use of HP-100 resin in the purification process resulted in a significant enrichment of CBD content, which was 4.2 times higher than that of the crude extract, with a recovery rate of 83.13%. The MIC against *S. aureus* was found to be 5 mg/mL, while no inhibitory effect was observed against *E. coli*. The IC50 values for the DPPH and ABTS assays were 0.1875 mg/mL and 2.988 mg/mL, respectively, indicating the potent antioxidant capacity of CBD. Additionally, CBD demonstrated a strong iron-reducing ability.

**Conclusion:**

These findings contribute to the development of CBD for broader applications in various industries, highlighting its potential as a valuable compound in health and wellness sectors.

## 1 Introduction

*Cannabis Sativa* L., a perennial dioecious herb belonging to the class Magnoliopsida, order Urticales, and the genus Cannabis, is indigenous to Central and East Asia (Vozza Berardo et al., 2024). This versatile plant has been used for millennia for its fibers, seed oil, and cannabinoids, which are rich in a variety of beneficial natural ingredients (Werf et al., 2010). The hemp derived from *C. Sativa* is now extensively utilized cross diverse sectors, including food, textiles, medicine, cosmetics, and light and chemical industries, due to its rich pharmacological activities. These properties include anti-epileptic and anti-convulsant effects (Alexander, 2016), neuroprotective actions (Tikoo et al., 2012), antiemetic capabilities (Whiting et al., 2015), analgesic effects (Bonini et al., 2018), as well as antibacterial and anti-inflammatory activities (Saxena, 2009). The biological properties of hemp is predominantly ascribed to its cannabinoid constituents. Among these, Cannabidiol (CBD) stands out as a major non-psychoactive cannabinoid with a broad spectrum of pharmacological effects, such as anti-tumor (Bonini et al., 2018), neuroprotection (Rong et al., 2017), metabolic regulation, immune modulation, anti-inflammatory (Robaina Cabrera et al., 2021), antibacterial and antioxidant (Mechoulam et al., 2002) activities.

In the realm of phenol extraction, a multitude of techniques have been developed, encompassing microwave extraction (Dhanani et al., 2017), ultrasound-assisted extraction (Afroz Bakht et al., 2019), supercritical fluid extraction (Joana Gil-Chávez et al., 2013), enzymatic hydrolysis (Gu et al., 2021), and deep eutectic solvent extraction (Nishijo et al., 2021).

However, for this study, the traditional heat reflux extraction method was selected due to its straightforward extraction apparatus, uncomplicated operation, and cost-effective nature (Moldovan et al., 2019). The underlying principle of this method involves the disruption of plant cell walls through sustained heating, facilitating the diffusion of the desired components into the solvent. Response surface methodology (RSM), as a robust statistical tool, was employed to optimize experimental variables, thereby enhancing the yield of bioactive compounds while minimizing the number of experimental runs (Zhong and Qiang, 2010; Pandey et al., 2018). In the quest for an optimal enrichment technique, macroporous resin was chosen over alternative methods such as polyamide chromatography, silica gel column chromatography, preparative high-performance liquid chromatography (HPLC), and high-speed counter-current chromatography (Wang et al., 2013). In the quest for an optimal enrichment technique, various methods such as polyamide chromatography, silica gel column chromatography, preparative high-performance liquid chromatography (HPLC), and high-speed counter-current chromatography were considered. Ultimately, macroporous resins were chosen as the preferred option due to their several advantageous properties. Macroporous resins offer a substantial adsorption capacity, diverse selectivity towards different compounds, low equipment cost, and ease of operation (Li et al., 2018). Their large pore size allows for efficient diffusion of target molecules, while their specific surface area and pore structure enable tailored adsorption and desorption behaviors. Additionally, macroporous resins are readily available and can be easily scaled up for industrial applications. These factors make macroporous resins an ideal choice for the efficient and cost-effective enrichment of CBD from industrial hemp extracts.

The objective of this study was to extract and concentrate CBD from industrial hemp using heat reflux extraction followed by enrichment with macroporous resin. To achieve this, we employed a single-factor experiment combined with response surface methodology (RSM) to meticulously optimize the CBD extraction process. This approach allowed us to identify and refine the critical parameters influencing extraction efficiency, resulting in a significant increase in the yield of CBD compared to traditional methods. Subsequently, employing CBD content as the dependent variable, we further refined the extraction parameters through RSM. A comparative analysis was then conducted on ten distinct resins to discern the parameters influencing their adsorption and desorption efficacy concerning the total CBD content. This process optimized the parameters to ensure the highest enrichment efficiency. Furthermore, the bioactivity of the enriched CBD was evaluated to establish a scientific foundation for the further utilization of industrial hemp resources.

## 2 Materials and methods

### 2.1 Materials and chemicals

Industrial hemp was sourced from the Heilongjiang Province cultivation base, qinggang, Heilongjiang Province. The specific variety of hemp was Cannabis sativa L., which is widely cultivated for its industrial applications due to its low THC content. The hemp plants were harvested in July 2023. The plant material was then dried and powdered before being used for CBD extraction.

The Beijing Beinachuang United Biotechnology Research Institute procured the CBD standard product. Chemicals including 1,1-diphenyl-2-picrylhydrazyl (DPPH), 2,2’-azino-bis(3-ethylbenzothiazoline-6-sulfonic acid) (ABTS), and vitamin C (Vc) were acquired from Sigma Aldrich (Shanghai) Trading Co., LTD. Analytical-grade reagents such as potassium ferricyanide (K_3_[Fe(CN)_6_]), trichloroacetic acid (TCA), anhydrous ferric chloride, and anhydrous sodium sulfate were supplied by Shantou Xilong Scientific Co., Ltd. Solvents like anhydrous ethanol, HPLC-grade acetonitrile, and formic acid were all obtained from Tianjin Komio Chemical Reagent Co., Ltd. The water utilized in our experiments was deionized and further purified using a Milli-Q academic water purification system (Millipore Corporation, Bedford, MA, USA).

A range of macroporous resins, including AB-8, D-101, HPD-100, HPD-300, HPD-400, HPD-600, HPD-826, NKA-9, X-5, and DM-130, was procured from Shihuida Chemical Co., Ltd. **Table 1** presents the physical properties of these resins. Each resin was immersed in a 95% ethanol solution overnight. On a subsequent day, they were thoroughly rinsed with deionized water in a continuous process until the effluent was clear of white turbidity and free of any ethanol odor.

**Table 1:** Physical characteristics of selected macroporous resins.

Name	polarity	Surface area (m^2^/g)	Average pore diameter (nm)	Particle diameter (mm)
NKA-9	polar	250–290	15.5–16.5	0.30–1.25
HPD-600	polar	550–600	8	0.30–1.20
HPD-826	Middle-polar	500–600	9.0–10.0	0.30–1.25
HPD-400	Middle-polar	500–550	7.5–8.0	0.30–1.20
HPD-300	Middle-polar	800–870	5.0–5.5	0.30–1.20
AB8	Weak-polar	480–520	13.0–14.0	0.30–1.25
DM-130	Weak-polar	500–550	9.0–10.0	0.30–1.25
D-101	Non-polar	480–520	25.0–28.0	0.30–1.25
X-5	Non-polar	500–600	29.0–30.0	0.30–1.25
HPD-100	Non-polar	650–700	8.5–9.0	0.30–1.25

### 2.2 Extraction and determination of CBD

The crushed industrial hemp (0.5 g) was precisely weighed and sieved through a 60-mesh sifter. The extraction process was meticulously conducted under predefined conditions, including temperature, time, liquid to solid ratio, ethanol concentration, and the number of extraction cycles. Subsequently, the resulting extracts were subjected to centrifugation at 3000 rpm for 5 min to separate the phases effectively. The supernatant was then filtered through a 5-micrometer membrane, and the concentration of CBD in the crude extract was quantified using HPLC.

### 2.3 Experimental design

#### 2.3.1 Single-factor experimental design

In accordance with the extraction conditions outlined in section 2.2, the extraction efficiency of CBD from industrial hemp was utilized as the primary metric for a single-factor experimental design. A series of single-factor experiments were conducted to investigate the impact of various parameters on CBD extraction yield. These parameters included extraction temperature (40-80°C), extraction time (30-150 min), ethanol concentration (20-100%), the liquid to solid ratio (10-30 mL/g), and the number of extraction cycles (1-5). Each variable was adjusted individually while maintaining others at a constant level. The concentration of CBD in each experimental group was quantified using HPLC.

#### 2.3.2 RSM design and statistical analysis

**Table 2** presents the selection of four critical factors (X_1_: extraction temperature, X_2_: extraction time, X_3_: ethanol concentration, X_4_: the liquid to solid ratio) that were identified as having the most significant impact on the experimental outcomes. These factors were systematically investigated using a BBD with three coded levels (-1, 0, 1) to optimize the CBD extraction process.

**Table 2:** Experimental variables and design levels for response surface methodology.

Variables	Code symbols	–1	Levels 0	1
Extraction time (min)	X_1_	30	60	90
Extraction temperature (°C)	X_2_	60	70	80
Ethanol concentration (%)	X_3_	60	80	100
Liquid-to-solid ratio (mL/g)	X_4_	20	25	30

The resulting experimental data were subjected to a comprehensive analysis through multiple regression with the Design-Expert software (Version 8.0.5.0, STAT-Ease, Minneapolis, USA). A second-order polynomial model was employed to elucidate the interactions between the independent variables and the dependent response variable (the CBD extraction rate). Furthermore, an analysis of variance (ANOVA) was conducted on the model’s fitting data, which facilitated the identification of the optimal conditions for CBD extraction.

### 2.4 Enrichment experiment

#### 2.4.1 Static adsorption and desorption tests

The optimal resin was selected through an evaluation of both adsorption and desorption capacities. A selection of pre-treated resins, namely AB-8, D-101, HPD-100, HPD-300, HPD-400, HPD-600, HPD-826, NKA-9, X-5, and DM-130, were each weighed to 0.3 g. Subsequently, 10 mL of the CBD sample solution was added to these resins. The mixtures were agitated at a rate of 120 rpm and allowed to adsorb for a duration of 24 h at a temperature of 25°C. Following this, 50 mL of deionized water was utilized to thoroughly rinse away impurities from each resin. Subsequently, a 70% ethanol solution (50 mL) was employed for desorption, which was carried out under the same agitation conditions for another 24 h at 25°C.

The CBD content in both the adsorbed and desorbed solutions was determined using HPLC. The adsorption and desorption capacities were calculated using the following formulas:


(1) $${\text{Q}}_{\text{e}}=({\text{C}}_{0}-{\text{C}}_{\text{e}})\times \frac{{\text{V}}_{0}}{\text{W}}$$

(2) $${\text{Q}}_{\text{d}}=\frac{{\text{C}}_{\text{d}}\times {\text{V}}_{\text{d}}}{\text{W}}$$

(3) $$\text{D}=\frac{\text{Cd}\times \text{Vd}}{({\text{C}}_{0}-{\text{C}}_{\text{e}})\times {\text{V}}_{\text{i}}}\times 100\%$$

Where Q_e_ and Q_d_ represent the adsorption and desorption capacities per gram of resin, respectively (mg/g). C_0_, C_e_, and C_d_ denote the initial, adsorption equilibrium, and desorption concentrations of CBD in the sample solution, respectively (mg/mL). V_0_ and V_d_ are the volumes of the initial and desorption solutions, respectively (mL). W is the dry weight of the macroporous resin (g), and D is the desorption rate (%).

#### 2.4.2 Adsorption kinetics study

A pre-treated batch of HPD-100 resin (2.0 g) was thoroughly mixed with a 20 mL volume of CBD sample solution. Throughout the adsorption process, the CBD content in the solution was meticulously monitored during the adsorption process, then a kinetic curve of HPD-100 adsorbing CBD was plotted.

#### 2.4.3 Adsorption isotherms

The influence of initial concentration and temperature on the adsorption of CBD by HPD-100 macroporous resin was systematically investigated. Specifically, 0.2 g portions of the resin were individually introduced into 10 mL aliquots of CBD sample solutions with varying concentrations. These mixtures were then subjected to agitation at temperatures of 25°C, 35°C, and 45°C for 5 h. The resultant data were analyzed and fitted to both the Langmuir and Freundlich isotherm models to determine the fit quality and derive pertinent parameters. The isothermal adsorption curves were constructed accordingly. The Langmuir equation, which assumes monolayer adsorption onto a homogeneous surface, is represented as follows:


(4) $${\text{Q}}_{\text{e}}={\text{Q}}_{\text{m}}\;{\text{K}}_{\text{L}}{\text{C}}_{\text{e}}/(1+{\text{K}}_{\text{L}}{\text{C}}_{\text{e}})$$

Where Q_e_ is the adsorption capacity per gram of resin (mg/g), C_e_ is the equilibrium concentration of CBD in the solution (mg/mL), and Q_m_ is the theoretical maximum adsorption capacity. K_L_ is the Langmuir adsorption equilibrium constant (mL/mg), indicative of the affinity between the adsorbate and the adsorbent.

The Freundlich equation, which is an empirical model for heterogeneous surfaces, is given by:


(5) $${\text{Q}}_{\text{e}}={\text{K}}_{\text{F}}{\text{C}}_{e}^{1/\text{n}}$$

In this equation, K_F_ and n are constants related to the adsorption capacity and intensity, respectively. The constant n describes the trend of the isotherm.

### 2.5 Dynamic adsorption and desorption tests

A glass column, measuring 400 mm in length with an inner diameter of 15 mm, was wet-packed with the pre-treated HPD-100 macroporous resin, and the resin bed volume (BV) was 30 mL. A sample solution was then loaded onto the column at a controlled flow rate of 2 BV/h, equating to 5 BV of the solution being processed through the column. Throughout the adsorption phase, the concentration of CBD was measured at every 10 mL increment of the eluent. These measurements were utilized to construct the penetration curve, which graphically represents the adsorption dynamics of CBD onto the HPD-100 resin within the column.

Subsequently, in the desorption phase, the column was sequentially flushed with deionized water followed by ethanol solutions with concentrations ranging from 10% to 100%. This gradient elution was performed at a rate of 2 BV/h to systematically assess the desorption efficiency of various ethanol concentrations. The concentration of CBD in the eluate was determined to select optimal elution concentration.

### 2.6 *In vitro* antimicrobial activity

The minimum inhibitory concentration (MIC) of CBD enriched from industrial hemp was ascertained using the broth dilution technique outlined in the manual M11 and M100 of the Clinical and Laboratory Standards Institute (Ossa-Tabares et al., 2020). The study focused on two bacterial strains: *Staphylococcus aureus* (ATCC 25923) and *Escherichia coli* K88. Distilled water served as a negative control, while penicillin acted as the positive control.

Isolated colonies from the purified strains were inoculated into Luria-Bertani (LB) broth and incubated with shaking for 4-6 h at 37°C. Subsequently, the bacterial suspension was adjusted to a concentration of 1×10^9^ CFU/L. The enriched CBD solution was prepared in a 96-well microplate with a twofold serial dilution, and 100 µL of each bacterial strain was added to the wells containing varying concentrations of the CBD solution or distilled water. All experimental procedures were conducted under a biosafety level 2 laminar flow hood to ensure a sterile environment.

### 2.7 *In vitro* antioxidant activity

#### 2.7.1 DPPH free radical scavenging activity

The DPPH free radical scavenging activity was evaluated following the methodology detailed by Floegel et al (Floegel et al., 2011). The procedure is summarized as follows: 100 μL of the sample solution and 100 μL of DPPH in ethanol solution (5 mmol/L) were combined in a 96-well plate. The mixture was vigorously shaken and then incubated at 25°C for 30 min in the dark. Subsequently, the absorbance was measured at a wavelength of 517 nm. Vc was employed as a positive control, and each experimental group was conducted in triplicate. The clearance rate of the DPPH radicals was calculated using the formula (GBD 2017 Diarrhoeal Disease Collaborators, 2020):


$$\text{clearance rate }(\%)=[1-({\text{A}}_{\text{s}}-{\text{A}}_{\text{b}})/{\text{A}}_{\text{c}}]\times 100\%$$

In this formula, A_s_ represents the absorbance of the reaction system consisting of the sample and DPPH solution. A_b_ denotes the absorbance of the DPPH solution without the sample, prepared by mixing DPPH with anhydrous ethanol. A_c_ is the absorbance of the negative control, which is a mixture of anhydrous ethanol and DPPH solution.

#### 2.7.2 ABTS free radical scavenging activity

The ABTS radical scavenging capacity of CBD samples was assessed using a spectrophotometric method, modified from the procedure reported by Li et al (Li et al., 2015). An ABTS solution at a concentration of 7.4 mmol/L was combined with an aqueous solution of potassium persulfate at 140 mmol/L and allowed to react in the dark at room temperature for 12 h. The resulting ABTS radical solution was then diluted to an absorbance of 0.7 ± 0.02 at 734 nm, serving as the ABTS stock solution.

Five different concentrations of enriched CBD products were prepared in anhydrous ethanol to create sample solutions, with an equal concentration of Vc solution serving as the positive control. A mixture was prepared by combining 0.8 mL of ABTS stock solution with 0.2 mL of each sample solution. This mixture was vortexed for 10 s and then incubated for 6 min. The absorbance was measured at 734 nm and denoted as A. This measurement was performed in triplicate for each sample. For the control group, deionized water was used in place of the sample solution, and the resulting absorbance was recorded as A_0_. The ABTS free radical scavenging rate was calculated using the following formula:


$$\text{clearance rate }(\%)=({\text{A}}_{0}-\text{A})/\text{A}\times 100\%$$

#### 2.7.3 Determination of iron reducibility

The iron reducibility of the sample was determined following the protocol outlined by Oyaizu (Oyaizu, 1986). The working solution for the reduction assay was prepared by thoroughly mixing 2.5 mL of K_4_Fe(CN)_6_ with 2.5 mL of a Na_2_HPO_4_ buffer solution at pH 6.6. To this mixture, 1 mL of the sample solution was added and the resulting solution was incubated at 50°C for 20 min. Subsequently, 2.5 mL of a 10% C_2_HCl_3_O_2_ solution was introduced to the mixture to achieve acidification. Afterward, 0.1 mL of a 0.1% FeCl_3_ solution and 2 mL of distilled water were added to the reaction mixture. The absorbance of the solution was measured at 700 nm, and this value was utilized as an indicator of the iron-reducing capacity of the extract.

### 2.8 HPLC analysis

A Shimadzu LC-10AVP Plus liquid chromatography system was employed to analyze the presence and concentration of CBD both before and after the enrichment process. The chromatographic conditions were optimized as follows: A C18 analytical column (4.6 mm × 150 mm, 5 μm), provided by Dikma Technology Co., Ltd., was utilized. The mobile phase consisted of 0.1% formic acid in water (A) and acetonitrile (B), maintained at a temperature of 25°C. The flow rate was set at 1.0 mL/min, and the detection wavelength was fixed at 228 nm.

### 2.9 Statistical analysis

The experimental data were statistically analyzed using Design Expert 13 software and GraphPad Prism statistical software 7.0. To ensure robustness and reproducibility, each experiment was conducted at least three times, and the results were presented as the mean value ± the standard deviation (SD). Statistical significance was determined at the *p* < 0.05 level using one-way analysis of variance (ANOVA) followed by Tukey’s multiple comparisons test to identify significant differences between groups. Design Expert 13 was employed for the statistical design and optimization of the RSM7. This software facilitated the evaluation of the response to regression equations, determination of the contribution and significance of the parameters, generation of the response surface plots, and determination of the best extraction strategy.

## 3 Results and discussion

### 3.1 Influence of single-factor variables on CBD extraction efficiency

**Figure 1:**
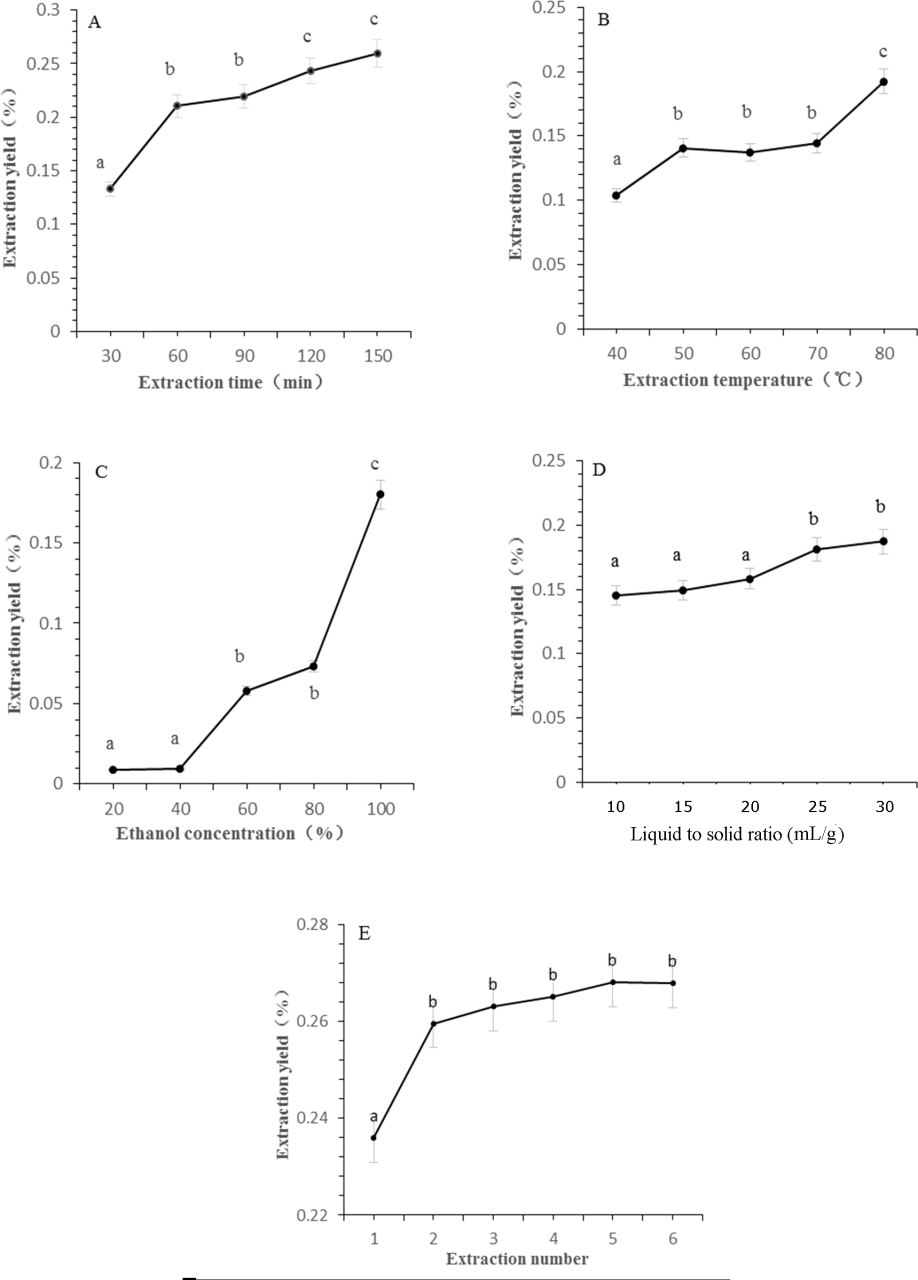
The effect of different extraction parameters on extraction yield of CBD. **(A)** Extraction time, **(B)** extraction temperature, **(C)** ethanol concentration, **(D)** liquid to solid ratio, **(E)** extraction cycles. Significant differences extraction parameters on extraction yield of CBD are indicated by different letters. Significance level was set as p < 0.05.

The efficiency of CBD extraction is intricately linked to the extraction parameters, which include duration, temperature, solvent concentration, and the liquid-to-solid ratio. Extended extraction durations facilitate prolonged solvent-sample contact, thereby enhancing extraction efficiency. However, excessively long periods may result in compound degradation due to temperature elevation and consequently increase both energy expenditure and processing time (Tomšik et al., 2016). **Figure 1A** illustrates the impact of reflux time on CBD extraction rates. The rate of CBD extraction increased significantly as the extraction time was extended from 30 to 60 min. Beyond this threshold, the marginal increase in CBD extraction was minimal, suggesting an optimal balance between extraction efficiency and processing time. Therefore, an extraction time of 60 min was selected for subsequent experiments to ensure efficient CBD extraction without unnecessary prolongation.

**Figure 1B** exhibits a positive correlation between CBD extraction rates and reflux temperature, with the extraction rate peaking at 80°C. Statistical analysis confirmed a significant difference compared to temperatures ranging from 40 to 70°C, indicating the temperature’s critical role in CBD extraction. Within the 50 to 70°C range, no significant variances were observed in extraction rates, leading to the selection of 70°C for further investigation due to its balance between efficiency and energy conservation.

The principle of “like dissolves like” dictates that CBD, being relative non-polar, is more soluble in ethanol. An increase in ethanol concentration can disrupt the plant cell matrix, enhance solvent permeation, and improve CBD solubility (Simić et al., 2016). As depicted in **Figure 1C**, CBD content escalated with the progression of ethanol concentration from 40% to 80%, reaching a maximum yield when anhydrous ethanol was utilized as the extraction solvent. This underscores the importance of solvent selection in the extraction process.

The liquid-to-solid ratio is another critical parameter in the extraction process, as it influences the availability of solvent for the extraction of CBD. **Figure 1D** shows that CBD yields increased in tandem with the liquid to solid ratio, with the rate of increase tapering off beyond a ratio of 25 mL/g. This suggests that an increased solvent volume per unit mass of plant material enhances extraction efficiency without causing dilution of the extract.

Extraction frequency is intrinsically linked to production efficiency and energy consumption in botanical extractions. While multiple extractions can maximize the yield of target components, they also escalate costs exponentially. As observed in **Figure 1E**, the most significant increase in CBD extraction rate occurred with two extraction cycles, leading to the adoption of this parameter as the optimal extraction condition.

### 3.2 Box-behnken design experimental results

#### 3.2.1 Model fitting and data analysis

Building upon the outcomes of the single-factor experiments, the BBD in conjunction with RSM was employed to further refine the optimization of the four independent variables. The response values are detailed in **Table 3**. A quadratic model was formulated to delineate the relationship between the response variable (Y: CBD content) and the independent factors, as follows:

**Table 3:** Box–Behnken experimental design matrix with four independent variables.

Exp.	X_1_/Extraction time (min)	X_2_/Extraction temperature (°C)	X_3_/Ethanol concentration (%)	X_4_/Liquid to solid ratio (mL/g)	Y/Extraction rate (%)
1	60	70	100	30	0.1427
2	30	70	60	25	0.1162
3	60	80	60	25	0.1610
4	60	60	60	25	0.1210
5	90	70	80	20	0.1428
6	90	60	80	25	0.1421
7	60	70	100	20	0.1603
8	60	80	80	30	0.1720
9	60	70	80	25	0.1765
10	90	80	80	25	0.2112
11	60	70	80	25	0.1755
12	60	60	100	25	0.1326
13	60	70	80	25	0.1793
14	90	70	60	25	0.1503
15	30	80	80	25	0.1736
16	30	60	80	25	0.1209
17	60	60	80	30	0.1155
18	30	70	80	30	0.1114
19	60	80	100	25	0.2279
20	60	70	60	30	0.1093
21	60	70	60	20	0.0955
22	90	70	100	25	0.1801
23	60	80	80	20	0.1664
24	30	70	80	20	0.1213
25	30	70	100	25	0.1530
26	60	60	80	20	0.1147
27	90	70	80	30	0.1565


$$\begin{array}{c} \text{Y}=0.1771+0.0156{\text{X}}_{1}+0.0304{\text{X}}_{2}+0.0203{\text{X}}_{3}+\\ 0.0005{\text{X}}_{4}+0.0041{\text{X}}_{1}{\text{X}}_{2}-0.0017\;{\text{X}}_{1}{\text{X}}_{3}+0.0059{\text{X}}_{1}{\text{X}}_{4}+\\ 0.0138{\text{X}}_{2}{\text{X}}_{3}+0.0012{\text{X}}_{2}{\text{X}}_{4}-0.0079{\text{X}}_{3}{\text{X}}_{4}-0.0119{\text{X}}_{1}^{2}\\ -0.0020{\text{X}}_{2}^{2}-0.0156{\text{X}}_{3}^{2}-0.0333{\text{X}}_{4}^{2} \end{array}$$

where X_1_, X_2_, X_3_ and X_4_ were the coded values of extraction time, extraction temperature, ethanol concentration, and the liquid-to-solid ratio, respectively.

The significance of the multiple regression model was assessed using Analysis of Variance (ANOVA). The findings are presented in **Table 4**. The model demonstrated statistical significance with a p-value significantly less than 0.0001 and an *F* value of 84.77 (**Table 4**). However, the *p*-value for the lack of fit, greater than 0.05, indicated that the model’s fit was not a concern, suggesting that it effectively captures the interplay between the CBD extraction rate and the four independent variables. The model determination coefficient (*R*²) was 0.9900, and the adjusted *R*² was 0.9783, underscoring the strong correlation between the predicted and experimental CBD extraction rates. This correlation substantiates the validity and applicability of the model established through the experimental design.

**Table 4:** ANOVA for the response surface quadratic model.

Source	Sum of squares	Degree of freedom	Mean square	*F*-value	*p*-value (Prob > *F*)
Model	0.0269	14	0.0019	84.77	<0.0001
X_1_	0.0029	1	0.0029	127.97	<0.0001
X_2_	0.0111	1	0.0111	490.43	<0.0001
X_3_	0.0049	1	0.0049	217.55	<0.0001
X_4_	3.413E-06	1	3.413E-06	0.1505	0.7048
X_1_X_2_	0.0001	1	0.0001	2.97	0.1107
X_1_X_3_	0.0000	1	0.0000	0.5403	0.4764
X_1_X_4_	0.0001	1	0.0001	6.14	0.0291
X_2_X_3_	0.0008	1	0.0008	33.72	<0.0001
X_2_X_4_	5.760E-06	1	5.760E-06	0.2540	0.6234
X_3_X_4_	0.0002	1	0.0002	10.87	0.0064
X_1_^2^	0.0008	1	0.0008	33.24	<0.0001
X_2_^2^	0.0000	1	0.0000	0.8944	0.3629
X_3_^2^	0.0013	1	0.0013	57.06	<0.0001
X_4_^2^	0.0059	1	0.0059	260.24	<0.0001
Residual	0.0003	12	0.0000		
Lack of fit	0.0003	10	0.0000	6.81	0.1347
Pure error	7.760E-06	2	3.880E-06		
Cor Total	0.0272	26			
*R* ^2^	0.9900				
Adj. *R* ^2^	0.9873				
Pred. *R* ^2^	0.9433				
Adeq Precision	35.0816				

#### 3.2.2 Model fitting and data analysis

**Figure 2:**
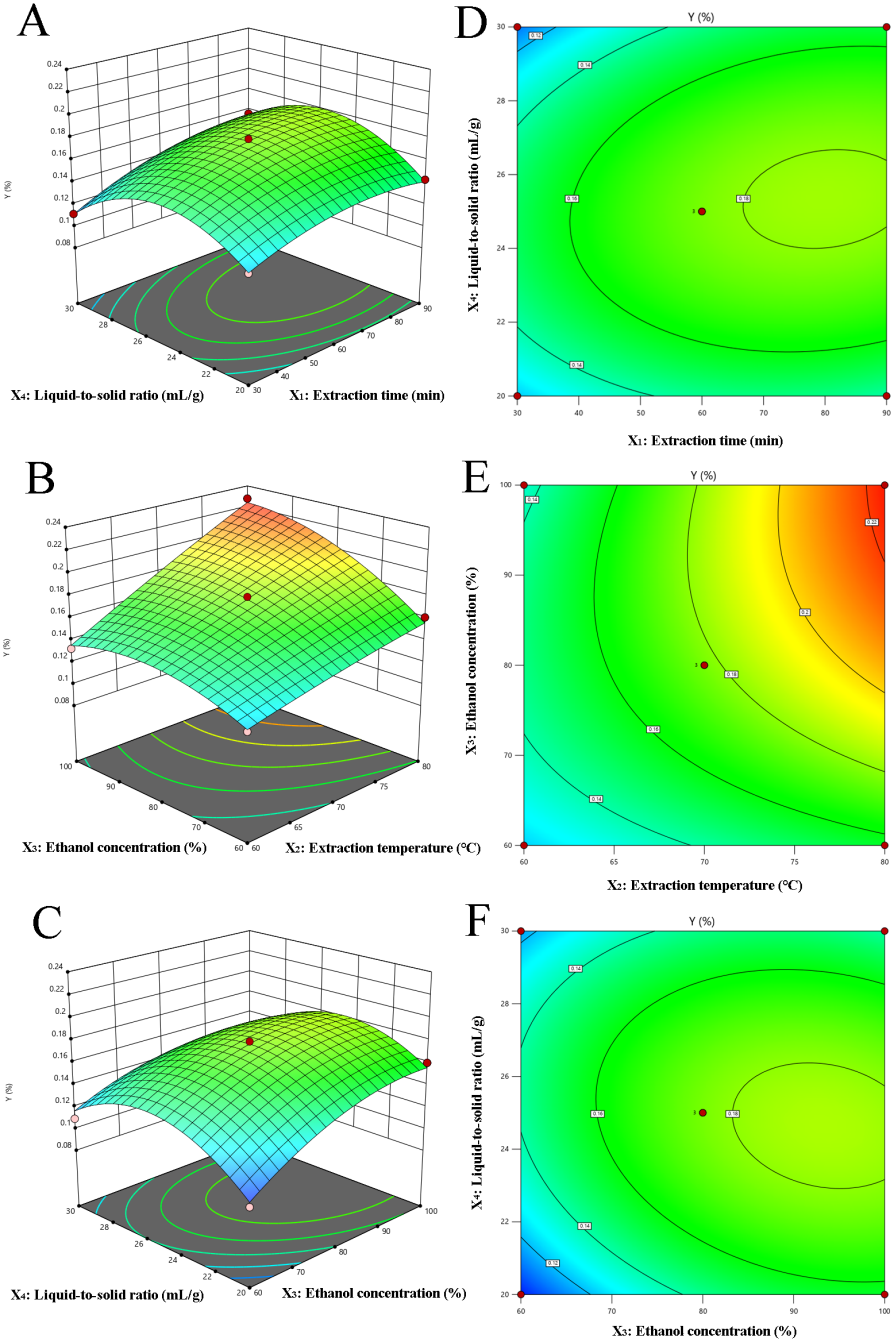
Response surface (3D) and contour plots (2D) depicting the effect of various extraction parameters (X_1_: extraction time, min; X_2_: extraction temperature, °C; X_3_: ethanol concentration, % and X_4_: the liquid to solid ratio, mL/g) on response Y. **(A–F)** corresponds to the different combinations of extraction time and liquid-to-solid ratio, extraction temperature and ethanol concentration, respectively.

The 3D response surface and corresponding 2D contour plots are shown in **Figure 2**, which indicates the interplay between variables in the CBD extraction process. Specifically, these plots highlight the interactions between two variables while holding the others constant. **Figures 2A, D** revealed the influence of extraction time (X_1_) and liquid-to-solid ratio (X_4_) on CBD yield. The CBD content demonstrates an upward trend at a constant liquid-to-solid ratio as extraction time extends from 30 to 80 min, followed by a slight decline. This suggests that while increased extraction time enhances CBD extraction, there is a threshold beyond which the benefits diminish. Similar trends were discernible in the interaction between ethanol concentration (X_3_) and liquid-to-solid ratio (X_4_), as depicted in **Figures 2C, F**. **Figures 2B, E** offered a composite view of the extraction temperature (X_2_) and ethanol concentration (X_3_) on CBD yield, with extraction time (X_1_) and liquid to solid ratio (X_4_) maintained at the level of 0. The data indicate that CBD yield is positively correlated with both increasing extraction time and liquid to solid ratio. Notably, the interaction between extraction time and liquid-to-solid ratio is of significant importance. The response surface analysis underscores the pivotal role of extraction time and liquid-to-solid ratio in the CBD extraction process. Other interactions, deemed inconsequential to the extraction efficiency, have not been included in this presentation, emphasizing the focus on key variables that truly impact the outcome.

Utilizing Design-Expert 10.0 software, the optimal parameters for CBD extraction were determined as follows: an extraction time of 85.31 min, a temperature of 79.54°C, an ethanol concentration of 98.17%, and a liquid-to-solid ratio of 24.43 mL/g. Considering the precision limitations of the equipment, these parameters were rounded to practical values for experimental purposes: 85 min for extraction time, 80°C for extraction temperature, and 98% for ethanol concentration. The actual extraction rate achieved was 0.23 ± 0.02%, which is near the predicted value of 0.23%. This demonstrates that the RSM, based on the BBD, is an effective tool for optimizing the CBD extraction process.

### 3.3 Enrichment studies

#### 3.3.1 Evaluation of static adsorption and desorption on resins

**Figure 3:**
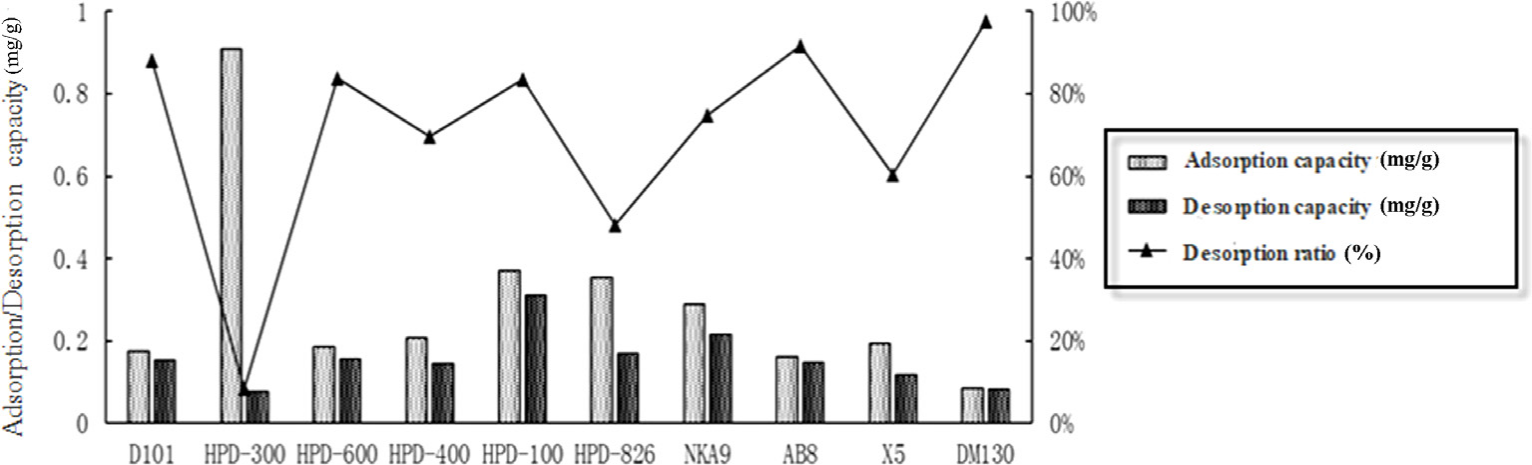
Evaluation of static adsorption, desorption capacities, and desorption ratios for CBD using ten different resins.

Several macroporous resins were evaluated for their adsorption and desorption capabilities to determine the most suitable one for CBD enrichment. The initial assessment focused on ten different resins: AB-8, D-101, HPD-100, HPD-300, HPD-400, HPD-600, HPD-826, NKA-9, X-5, and DM-130. Each resin was subjected to static adsorption and desorption tests as described in the methods section. The results, presented in **Figure 3**, revealed that HPD-300, HPD-100, HPD-826, and NKA-9 demonstrated the highest adsorption capacities. However, HPD-300 had the lowest desorption rate, indicating that it would retain a significant amount of CBD and hinder efficient recovery. Similarly, resins like HPD-826, HPD-400, and X-5 also exhibited suboptimal desorption characteristics.

HPD-100 resin stood out as the most suitable option due to its balanced performance in both adsorption and desorption. It possessed a high adsorption capacity of 0.37 mg/g while maintaining a desorption rate of 83.55%, ensuring effective recovery of CBD during the purification process. This balance between adsorption and desorption efficiency is crucial for achieving a high yield of purified CBD with minimal losses.

Furthermore, the HPD-100 resin offered several additional advantages. Its non-polar nature facilitated selective adsorption of CBD while excluding polar impurities. Additionally, its moderate pore size and surface area provided adequate binding sites for CBD molecules without causing excessive diffusion limitations. These properties contribute to the overall efficiency and effectiveness of the CBD enrichment process.

Based on this comprehensive evaluation, HPD-100 resin was selected for further investigation in the dynamic adsorption and desorption studies and subsequent optimization of the purification process.

#### 3.3.2 Influence of pH on the adsorption capacity of HPD-100

According to the previous findings, HPD-100 was identified as the resin of interest for further investigation into the impact of pH on CBD adsorption capacity. Four 10 mL aliquots of CBD solution were adjusted to pH values of 1.0, 3.0, 5.0, and 7.0, and to each, 1.0 g of HPD-100 resin was added. These mixtures were then agitated at 120 rpm for 6 h at 25°C. Once adsorption equilibrium was reached, the CBD content in the solutions was measured to determine the resin’s adsorption capacity.

The pH level plays a pivotal role in the ionization of solutes, thereby affecting their interaction with the solution. As shown in **Table 5**, the adsorption capacity of HPD-100 decreased as the pH increased, with optimal adsorption observed at a pH of 3. This suggests that the ionization state of CBD and the resin at different pH levels affects their affinity for each other, which is an important consideration for the adsorption process.

**Table 5:** Influence of pH on the adsorption capacities of CBD by HPD-100 resin.

pH values	The adsorption capacity of CBD (mg/g)
1	0.01
3	0.30
5	0.20
7	0.17

#### 3.3.3 Adsorption kinetics on HPD-100 resin

**Figure 4:**
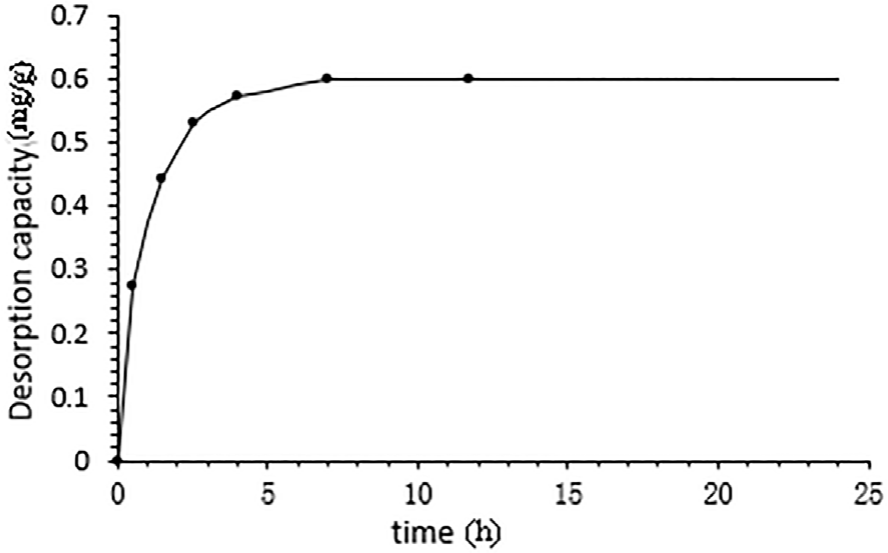
Kinetics profile of CBD adsorption onto HPD-100 resin.

**Figure 4** illustrates the adsorption kinetics of CBD onto HPD-100 resin over time. The adsorption capacity of CBD increased with the adsorption duration, reaching equilibrium after 8 h. This indicates that 8 h is the optimal time for HPD-100 to achieve adsorption equilibrium, which is a critical parameter for the practical application of the resin in the extraction process.

#### 3.3.4 Adsorption isotherm analysis on HPD-100 resin

**Figure 5:**
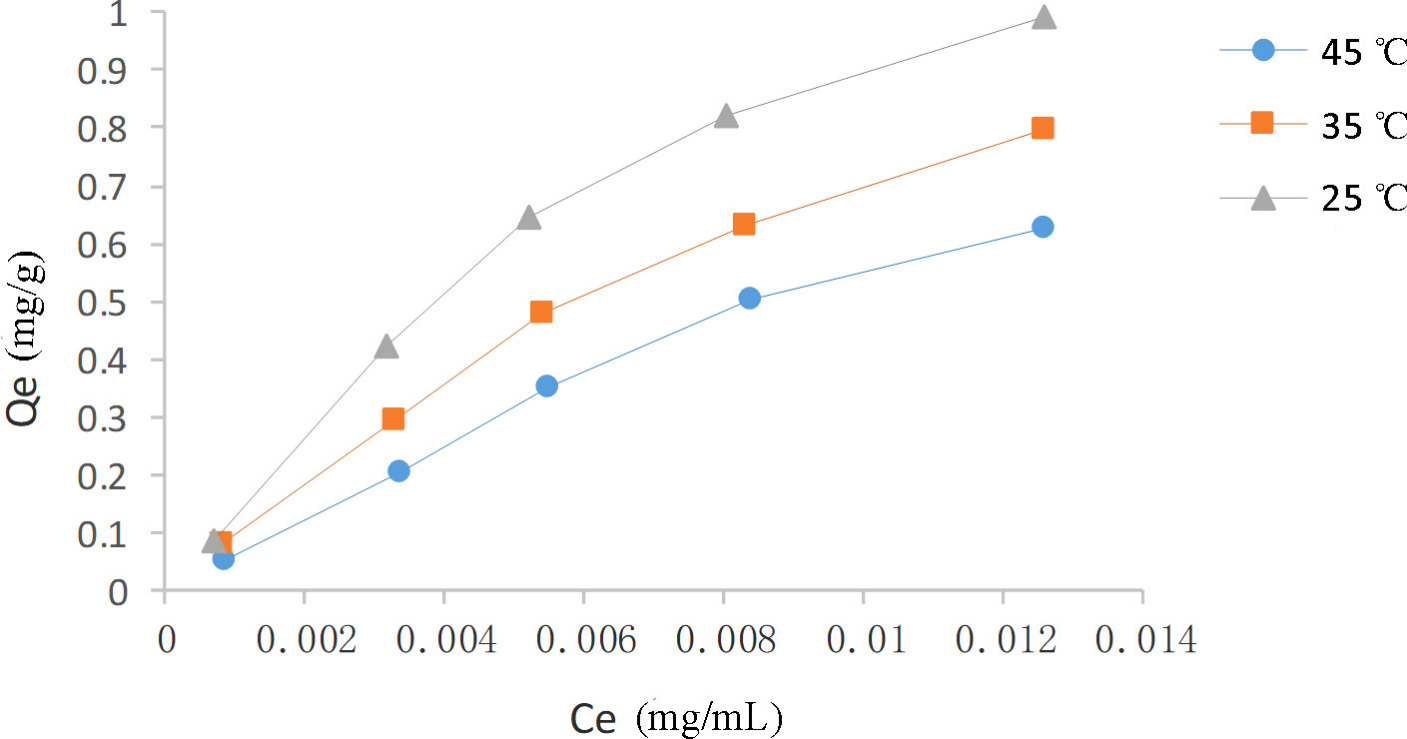
Adsorption isotherms of CBD on HPD-100 resin at temperatures of 25°C, 35°C, and 45°C.

The adsorption capacity of HPD-100 for CBD was found to be temperature-dependent, with a decrease in capacity observed as temperature increased, as shown in **Figure 5**. This trend indicates an exothermic adsorption process. Comparatively, 25°C was found to be more favorable for enhancing the adsorption efficiency of HPD-100 for CBD than 35°C and 45°C, and thus, 25°C was selected for the subsequent experiment. At an initial CBD concentration of 18.6 μg/mL, the adsorption capacity improved with increasing concentration, though the rate of increase slowed and approached saturation at a certain level.

Moreover, the Langmuir and Freundlich models were employed to analyze the adsorption data. The isotherm equations and parameters were presented in **Table 6**. The Langmuir isotherm assumed monolayer adsorption on a homogenous surface without interactions between adsorbed molecules. In contrast, the Freundlich isotherm describes non-ideal adsorption behavior within a specific concentration range (Wang and Wang, 2019). The high correlation coefficients for both the Langmuir (0.9918-0.9972) and Freundlich (0.9531-0.9818) equations, nearing unity, suggest that both models are suitable for characterizing the CBD adsorption process on HPD-100 resin. Additionally, the value of 1/n being less than 1 indicates favorable adsorption of CBD onto HPD-100 resin.

**Table 6:** Langmuir and Freundlich adsorption parameters at 25°C, 35°C, and 45°C.

Temperature (°C)	Langmuir equation	*R* ^2^	Qm (mg/g)	Freundlich equation	*R* ^2^	1/n
25	Ce/Qe=0.5384Ce+ 0.013	0.99182	1.86	Qe=21Ce0.7949	0.98183	0.7949
35	Ce/Qe=0.5574Ce+ 0.0087	0.99716	1.79	Qe=17.01Ce0.7076	0.95312	0.7076
45	Ce/Qe=0.5631Ce+ 0.0054	0.99415	1.77	Qe=16.67Ce0.6349	0.96644	0.6349

### 3.4 Dynamic adsorption and desorption studies on HPD-100 resin

**Figure 6:**
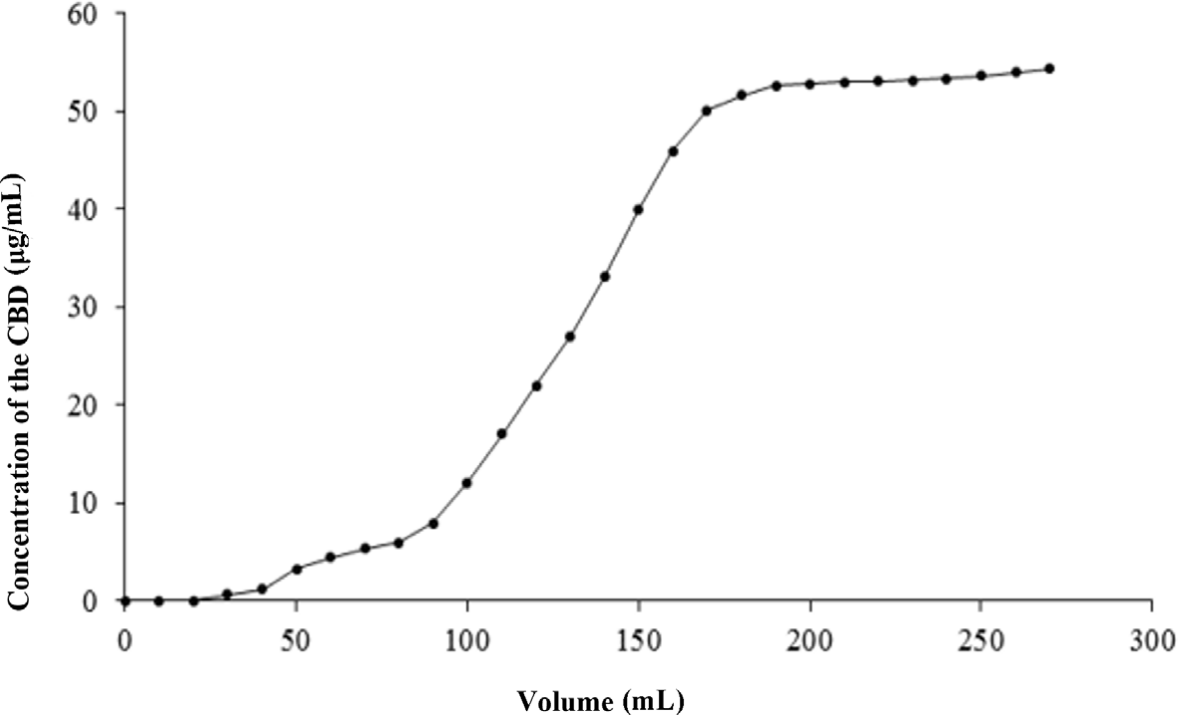
Dynamic breakthrough curves for CBD adsorption on HPD-100 resin.

The dynamic penetration curves were constructed based on the effluent volume and solute concentration data. As illustrated in **Figure 6**, the effluent concentration rapidly rose to 10% of the sample concentration after 2.6 BV, marking the onset of penetration (Sun et al., 2015). This increase continued until a stable plateau was reached at 12 BV. Consequently, the dynamic saturated adsorption volume for the CBD sample solution dynamically on the macroporous resin HPD-100 was determined to be 2.6 BV.

As presented in **Table 7**, the dynamic elution process involved using 5 BV of eluent for each ethanol concentration, with a gradient elution rate of 2 BV/h. Ethanol concentrations ranging from 0% to 30% were effective in eluting only a small amount of CBD along with numerous impurities. However, as the ethanol concentration in the eluent increased to 90%, the desorption volume reached its peak, providing a theoretical foundation for the industrial application of CBD in hemp. Therefore, at a flow rate of 2 BV/h, 30% ethanol was chosen for impurity elution, while 90% ethanol was employed for the desorption of CBD.

**Table 7:** Elution gradient results for the purification of CBD from HPD-100 resin.

Ethanol concentration (%)	CBD content (mg)	CBD recovery rate
0%	0	84.84%
10%	0.0821	
30%	0.0946	
50%	0.1265	
70%	0.1731	
90%	0.2822	
100%	0	

The optimized conditions for adsorption and desorption using HP-100 macroporous resin are as follows: an initial CBD sample solution concentration of 18.6 μg/mL, a pH of 1.0, absorption and desorption flow rates of 2 BV/h, and a temperature of 25°C. The elution process utilized 30% ethanol for impurity removal and 90% ethanol for CBD desorption. Under these conditions, the concentration of CBD was elevated to 1.19%, with a recovery rate of 83.13 ± 2.27%.

### 3.5 *In vitro* antimicrobial assessments

The enriched CBD demonstrated significant antibacterial activity against *S. aureus* with an MIC of 5 mg/mL. However, it displayed no discernible antibacterial effect against *E. coli*. This selective antibacterial property suggests potential therapeutic applications of CBD in targeted antimicrobial treatments.

### 3.6 *In vitro* antioxidant assessments

#### 3.6.1 DPPH scavenging activity

**Figure 7:**
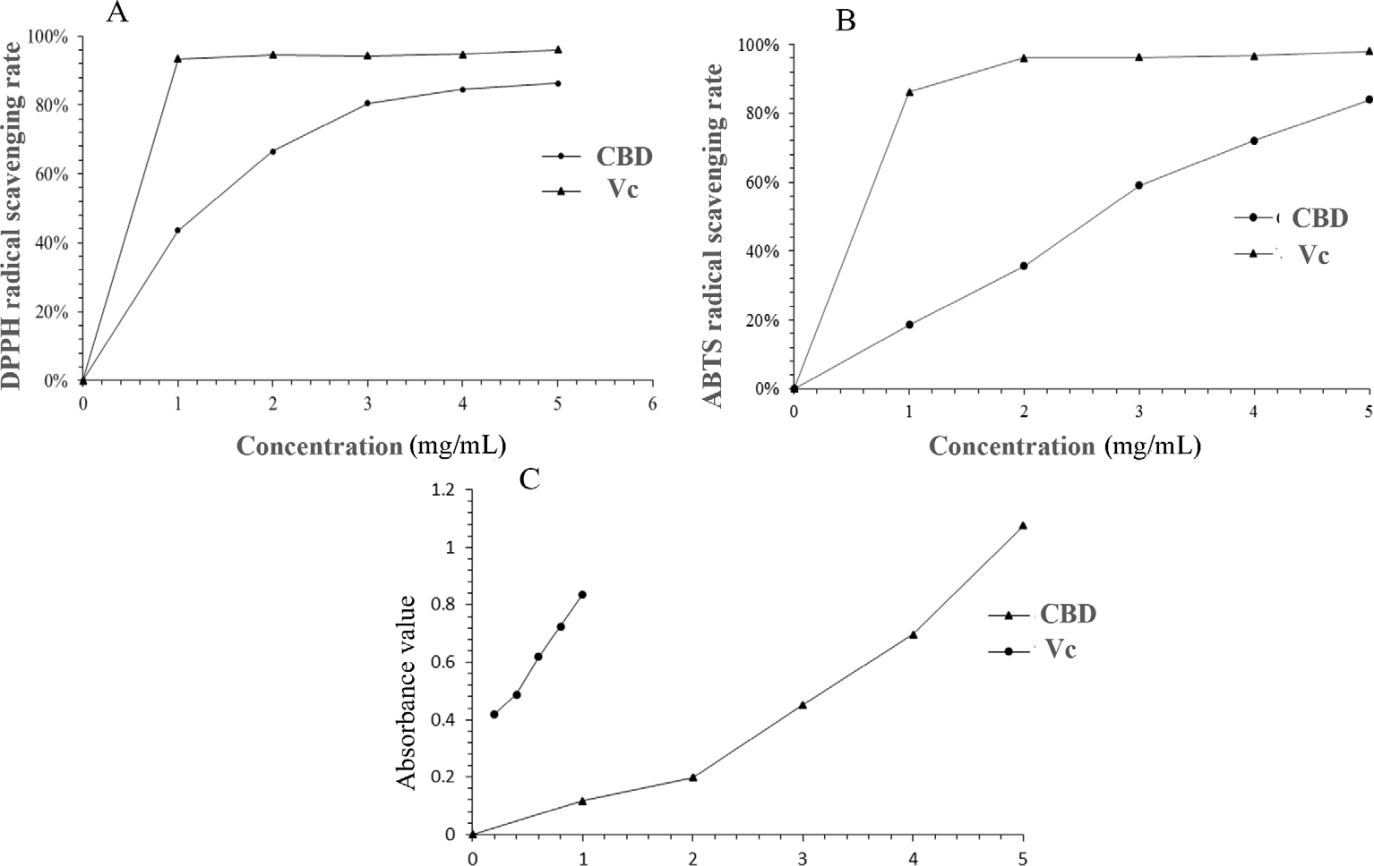
Comparative analysis of DPPH radical scavenging activity **(A)**, ABTS radical scavenging activity **(B)**, and reducing power **(C)** between CBD-enriched extracts and Vc.

DPPH, a stable organic free radical at room temperature, can be reduced, causing a shift from a dark purple to a colorless or light yellow solution (Xing et al., 2020). As depicted in **Figure 7A**, the DPPH radical scavenging capacity of the enriched CBD was dose-dependent, with an IC50 value of 1.856 mg/mL. This indicates that the enriched CBD possesses the ability to scavenge DPPH radicals, albeit with a weaker antioxidant effect compared to Vc.

#### 3.6.2 ABTS radical scavenging activity

**Figure 7B** illustrated the ABTS radical scavenging rate of the enriched CBD, which followed a linear relationship equation with the concentration (y = 0.1723x + 0.0178, R^2^ = 0.9901). The IC50 value was determined to be 2.988 mg/mL. At a sample concentration of 5 mg/mL, the maximum scavenging rate reached 83.88%, confirming the enriched CBD*’*s robust ABTS radical scavenging capability.

#### 3.6.3 Reducing power analysis

The reducing power analysis serves as a valuable indicator of a substance*’*s antioxidant potential. Substances with strong reducing capabilities can inhibit the chain reaction of free radicals, and their antioxidant efficacy is proportional to the sample*’*s absorbance. **Figure 7C** compared the reducing power of the enriched CBD with that of Vc. At a concentration of 5 mg/mL, the absorbance of the sample was 1.08, demonstrating a concentration-dependent relationship. Nevertheless, Vc exhibited a stronger reducing ability than the enriched CBD at equivalent concentrations. These results provide valuable insights into the potential therapeutic applications of CBD in antioxidant formulations.

In conclusion, our comprehensive evaluation of the bioactivity of the enriched CBD, including *in vitro* antibacterial and antioxidant assays, provides valuable insights into the potential therapeutic applications of CBD across various industries.

## 4 Conclusion

In this experiment, the extraction and enriched conditions were optimized, and the potential antimicrobial and the antioxidant activities were investigated. The optimal extraction results exhibited that the predicted value of CBD yield was in agreement with the experimental value. In addition, the yield increased obviously after enrichment. The enriched CBD had an evident scavenging effect on DPPH, hydroxyl radical, and iron reducing power. And CBD had inhibitory effect on *S. aureus*. In conclusion, the above studies provided theoretical basis for the effective application of CBD in industrial hemp.

## Data Availability

The original contributions presented in the study are included in the article/supplementary material. Further inquiries can be directed to the corresponding author.
